# Termite colonies from mid-Cretaceous Myanmar demonstrate their early eusocial lifestyle in damp wood

**DOI:** 10.1093/nsr/nwz141

**Published:** 2019-09-13

**Authors:** Zhipeng Zhao, Xiangchu Yin, Chungkun Shih, Taiping Gao, Dong Ren

**Affiliations:** 1 College of Life Sciences, Capital Normal University, Beijing 100048, China; 2 Northwest Institute of Plateau Biology, Chinese Academy of Sciences, Xining 810008, China; 3 Department of Paleobiology, National Museum of Natural History, Smithsonian Institution, Washington, DC, 20013–7012, USA

**Keywords:** aggregation, Termitoidae, Stolotermitidae, cooperative brood care, caste

## Abstract

Insect eusociality is characterized by cooperative brood care, reproductive division of labour and multiple generations of adults within a colony. The morphological specializations of the different termite castes from Burmese amber were recently reported, indicating the termites possessed advanced sociality in the mid-Cretaceous. Unfortunately, all the reported Cretaceous termites are individually preserved, which does not cover the behaviours of the cooperative brood care and multiple generations of adults in the nests of the Cretaceous termites. Herein, we report three eusocial aggregations from colonies of the oldest known Stolotermitidae, *Cosmotermes***gen. nov.**, in 100 Ma mid-Cretaceous Burmese amber. One large aggregation, comprising 8 soldiers, 56 workers/pseudergates and 25 immatures of different instars, additionally presents the behaviours of cooperative brood care and overlapping generations. Furthermore, taphonomic evidence indicates *Cosmotermes* most probably dwelled in damp/rotting wood, which provides a broader horizon of the early societies and ecology of the eusocial *Cosmotermes*.

## INTRODUCTION

Insect eusociality, the most sophisticated level of animal sociality, is characterized by cooperative brood care, reproductive division of labour and multiple generations of adults within a colony [1,2]. Complex eusocial behaviours and nest dwelling enhance the survival and/or reproduction of termites. With wood-feeding, termites became one of the most successful insect taxa on Earth, with broad taxonomic diversity and high individual counts. Termites evolved from cockroaches and started an early radiation in the Early Cretaceous [3–8]. Until now, about 43 termite species have been reported in the Cretaceous and the most diverse species were reported from Burmese amber. Among the Cretaceous species, one of the oldest known termites hitherto is the wings of *Valditermes brenanae* found from Hauterivian England (≈130 Ma, the Lower Cretaceous) [9–11]. Besides, *Meiatermes bertrani* was found from the similar age of Barremian Spain [10,12,13]. Studies using fossil or molecular data suggest the divergence time of termites was in the Upper Jurassic [4,5,14,15].

Recently, the morphologically diverse castes of *Krishnatermes yoddha*, *Ginormotermes rex* and *Anisotermes xiai* in mid-Cretaceous have been reported from Burmese amber, which indicated the advanced sociality of the mid-Cretaceous termites [5,16,17], while the Cretaceous termites documented hitherto from compression fossils or amber are all individually preserved or only with isolated wings [10,18,19]. Consequently, the cooperative brood care and multiple generations of adults, the two behaviour traits for eusociality, of the Cretaceous termites have not been reported. Therefore, the eusocial aggregations of workers, soldiers and/or immatures are the key clues for tracing the inter-individual interactions and behaviours of the termites. The oldest termite eusocial aggregations hitherto with few workers and soldiers were of *Nasutitermes* (Termitidae) reported with an age of 17 Ma Miocene [2,20].

## RESULTS AND DISCUSSION

### Amber and specimens

**Figure 1.**
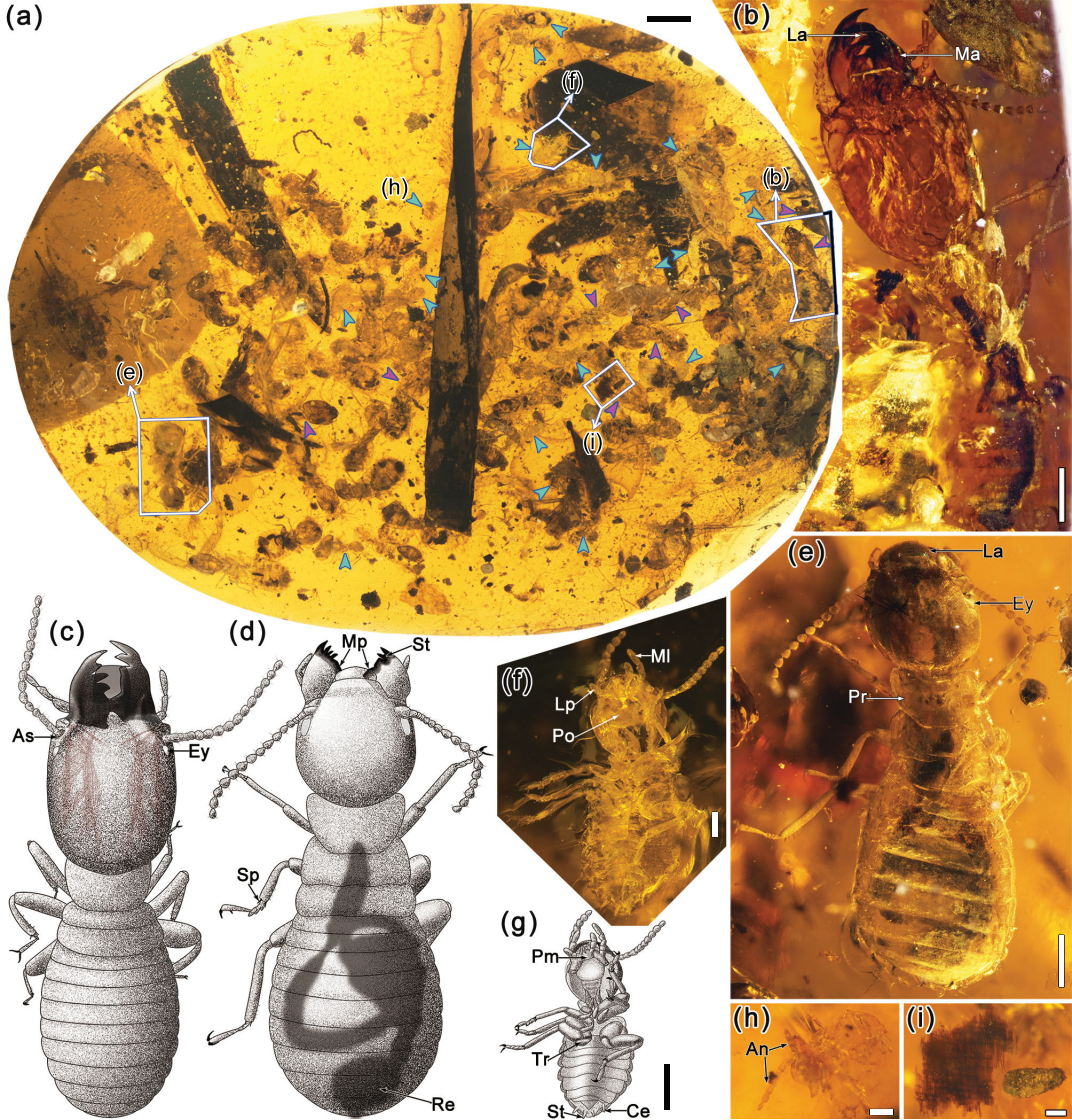
Photographs and drawings of *Cosmotermes multus***gen. et sp. nov.** in Burmese amber CNU008418. (a) Photograph of termite gregariousness in the amber. Among the 89 termites, the cyan arrows point to the immatures and the violet arrows point to the soldiers; other individuals are workers/pseudergates. The darker area on the left is modelling clay to enhance the visibility of some termites. (b) Photograph of holotype soldier (CNU–TER–BU–2018077) in dorsal view. (c) Reconstructive drawing of the soldier. Dark-red colour shows the visible muscles in the head. (d) Reconstructive drawing of the worker/pseudergate. Shadow shows the dimly visible intestine based on observations of all workers/pseudergates preserved. (e) Photograph of paratype worker/pseudergate (CNU–TER–BU–2018005) in dorsal view. (f) Photograph of paratype immature (CNU–TER–BU–2018037) in ventral view. (g) Drawing of the immature (CNU–TER–BU–2018037). (h) Photograph of paratype immature (CNU–TER–BU–2018041). (i) Dampwood preserved with *Cosmotermes multus* in CNU008418. Scale bars: 2 mm in (a); 0.5 mm in (b), (c) and (e)–(g); 0.2 mm in (d), (h) and (i). La, labrum; Ma, mandible; As, antenna socket; Ey, eye; Mp, molar plate; St, subsidiary tooth; Sp, spur; Re, rectum; Ml, maxillary palp; Lp, labial palp; Po, postmentum; Pm, prementum; Tr, trochanter; St, stylus; Ce, cercus; Pr, pronotum; An, antenna.

**Figure 2.**
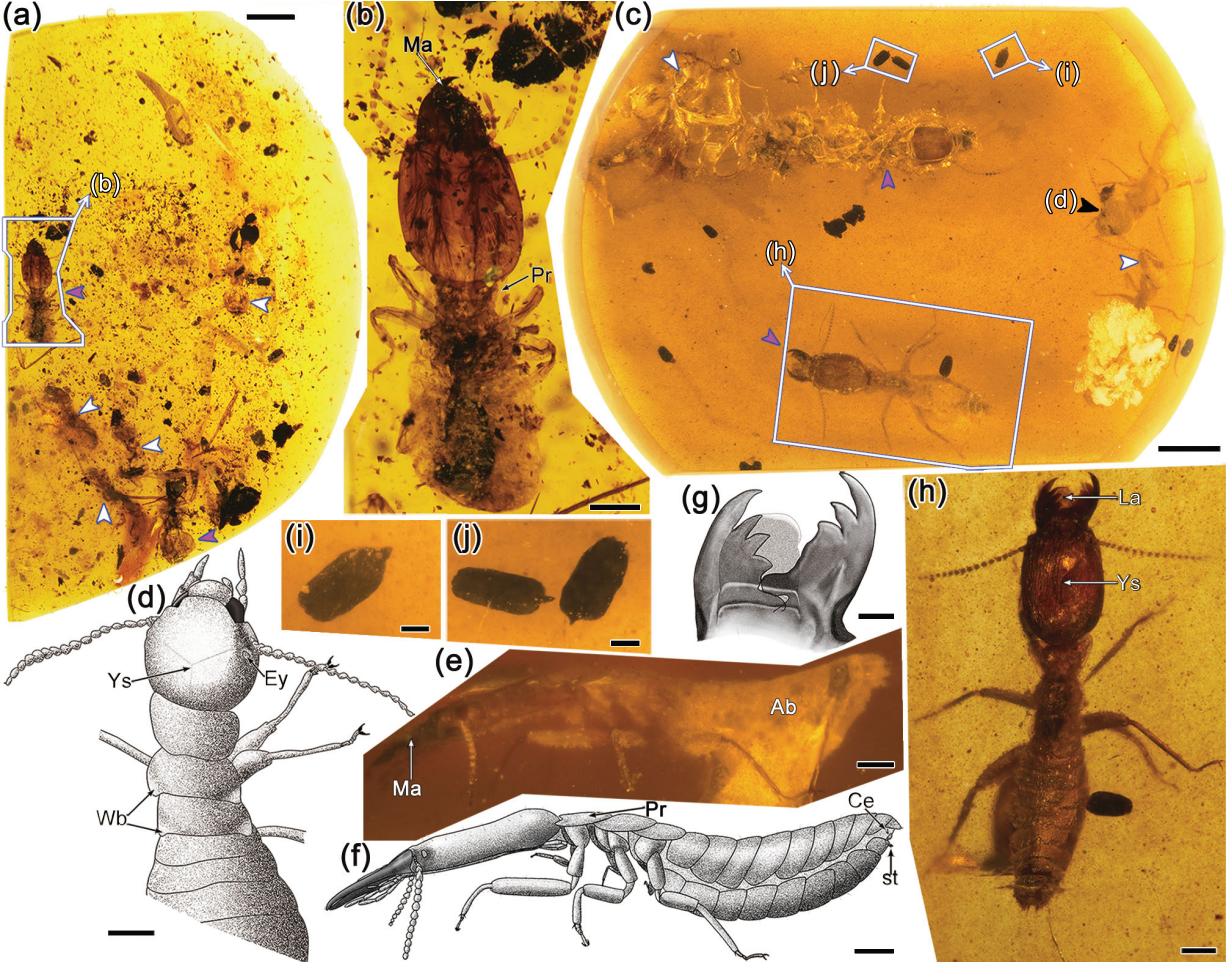
Photographs and drawings of *Cosmotermes multus* in CNU008281 (a) and (b) and *Cosmotermes opacus* in CNU008267 (c)–(j). (a) *Cosmotermes multus* paratypes in CNU008281. The violet arrows point to the two soldiers and the white arrows point to the four workers/pseudergates. (b) Photograph of *Cosmotermes multus* paratype soldier (CNU–TER–BU–2018101) in dorsal view. (c) Overview of *Cosmotermes opacus* paratypes in CNU008267; the violet arrows point to the soldiers, the white arrows point to the workers/pseudergates and the black arrow points to the nymph. (d) Drawing of the paratype nymph (CNU–TER–BU–2018202). (e) and (f) Photograph and reconstructive drawing of the holotype soldier (CNU–TER–BU–2018201) in lateral view. (g) Drawing of the mandibles of the holotype soldier (CNU–TER–BU–2018201). (h) Photograph of the holotype soldier (CNU–TER–BU–2018201) in dorsal view. (i) and (j) Rice-shaped fecal pellets preserved together with the termites. The surfaces of the fecal pellets are relatively smooth without distinct ridges. Scale bars: 2 mm in (a) and (c); 0.5 mm in (b), (d) and (h)–(j); 0.2 mm in (e)–(g). Ma, mandible; Pr, pronotum; Ey, eye; Ys, Y-suture; Wb, wing bud; Ab, abdomen; St, stylus; Ce, cercus; La, labrum.

**Figure 3.**
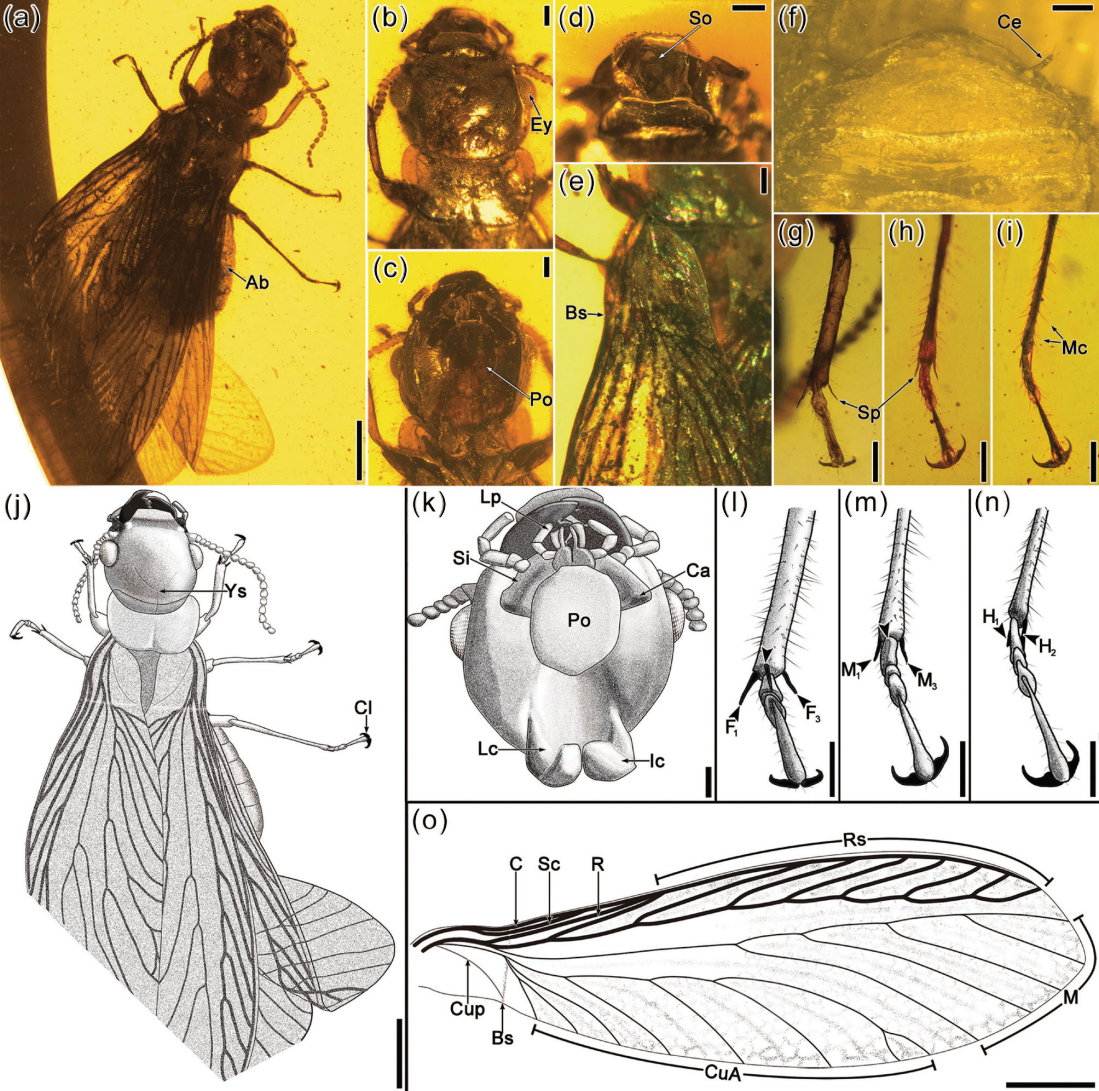
Photographs and drawings of *Cosmotermes opacus***sp. nov.**, imago (alate) CNU–TER–BU–2018206. (a) Dorsal habitus. (b) Head and pronotum in dorsal view. (c) Head and pronotum in ventral view. (d) Labrum and clypeus. (e) Left forewing base. (f) Postabdomen in ventral view. (g)–(i) Fore-, mid- and hind leg with tibia, tarsi and claws. (j) Dorsal habitus. (k) Head in ventral view. (l)–(n) Fore-, mid- and hind leg with tibia, tarsi and claws. (o) Reconstructive drawing of the forewing venation. Scale bars: 0.5 mm in (a), (j) and (o); 0.2 mm in others. Ab, abdomen; Ey, eye; Po, postmentum; So, subsidiary tooth; Bs, basal suture; Ce, cercus; Sp, spur; Mc, macrosetae; Ys, Y-suture; Cl, claw; Lp, labial palp; Si, stipe; Ca, cardo; Lc, lateral cervical sclerite; Ic, inner cervical sclerite; F, M and H with subscript respectively represent fore-leg tibial spurs, mid-leg tibial spurs and hind-leg tibial spurs.

In this research, four pieces of Burmese amber with three well-preserved termite aggregations and an individual alate were studied. All the amber samples were collected from Hukawng Valley, Kachin State, Northern Myanmar. The age was 98.79 ± 0.62 Ma, mid-Cretaceous (Cenomanian) [21–24]. The first amber piece of CNU008418 (Fig. 1a and Supplementary Figs 1a and 2) has a total of 89 termite individuals (*Cosmotermes multus***gen. et sp. nov.**), including eight soldiers, 56 workers/pseudergates and 25 immatures of different instars, preserved in the same inner layer (Supplementary Fig. 1b). Soldiers (e.g. Fig. 1b and c) are all with robust long mandibles and extended head capsules. Wingless workers/pseudergates have body lengths similar to those of soldiers but with rounded heads (e.g. Fig. 1d and e). The immatures of different instars (e.g. Fig. 1f–h) have obviously smaller body sizes, fewer antennomeres and weakly sclerotized exoskeletons. Besides the termites, fecal pellets, frass and a small piece of dampwood are also preserved in the same layer (e.g. Fig. 1i). The second amber piece CNU008281 (Fig. 2a) has six termites (*C. multus***gen. et sp. nov.**; Supplementary File 1), including two soldiers (e.g. Fig. 2b) and four workers/pseudergates (Supplementary File 1) in a layer and wood debris spreading around in the same layer. Embedded in the third amber piece CNU008267 (*Cosmotermes opacus***sp. nov.**; Fig. 2c and Supplementary File 1) are one nymph (Fig. 2d and Supplementary Fig. 3a, b, d and e), two workers/pseudergates (e.g. Supplementary Fig. 3c, f and g), two soldiers (e.g. Fig. 2e–h and Supplementary Fig. 3h–n) and several fecal pellets around the termites (e.g. Fig. 2i and j). Preserved in the fourth amber piece CNU008266 is a single termite alate (*C. opacus***sp. nov.**, CNU–TER–BU–2018206, Fig. 3).

### Systematic palaeontology

Order Blattodea, Brunner von Wattenwyl, 1882.

Epifamily Termitoidae, Latreille, 1802.

Family Stolotermitidae Holmgren, 1910.

Genus *Cosmotermes* Zhao, Yin, Shih & Ren **gen.** nov.

**Type species.** *Cosmotermes multus* **sp. nov.**

**Etymology.** The generic name is a combination of the Greek prefix ‘Cosmo-’, which means ‘orderly’, referring to termites living together in an orderly way, and ‘*termes*’ is a common suffix for generic name of termites. Gender masculine.

**Diagnosis.** See Supplementary Text for the genus diagnosis and type of specimen descriptions.

*Cosmotermes multus* Zhao, Yin, Shih & Ren **gen. et sp. nov.**

**Etymology.** The specific name ‘multus’ means multiple, referring to the multiple termite castes that are preserved together.

**Diagnosis.** Exoskeleton hyaline; head flat; antenna moniliform with 16–17 articles; ocelli absent; compound eyes vestigial with anterior emargination; Y-suture on head invisible; tibial spur formula 3–3–2; tarsi tetramerous; arolium absent; abdomen oval in dorsal/ventral view but flat in lateral view. *Worker*/*pseudergate*, right mandible with subsidiary tooth; pronotum width slightly narrower than head. *Soldier*, mandibles elongate, taking up about one-third of the length of the head, with incisive teeth, decurved from stem to apex.

**Holotype.** Soldier: CNU–TER–BU–2018077 (Fig. 1b) is housed at the Capital Normal University (CNU), a termite soldier, with head well preserved but other body structures partly preserved.

**Paratypes.** All termites listed in Supplementary File 1. Representatives: worker/pseudergate, CNU–TER–BU–2019005 (Fig. 1d and e), with the digestive tract slightly visible; Immatures, CNU–TER–BU–2018037 (Fig. 1f and g); Soldier, CNU–TER–BU–2018101 (Fig. 2

**Diagnosis.** Exoskeleton opaque; head flat; antennae moniliform with 18–20 articles; ocelli absent; compound eyes present; Y-suture present; tibiae slender; tibial spur formula 3–3–2; tarsi tetramerous; basitarsus elongate; arolium absent; cerci thin and short, with about five articles; styli present. *Imago*, head rounded dorsally, flat laterally; right mandible with subsidiary tooth; C, Sc R and Rs more sclerotized and pigmented than M and Cu; C not uniting with costal margin; basal suture straight on forewings; medial field covering wing apex, with about four branches; basal suture straight. *Worker/pseudergate*, compound eyes vestigial and small; abdomen oval. *Soldier*, mandibles elongate, taking up about one-third of the length of the head, with incisive teeth, decurved from stem to apex; abdomen spindly.

**Holotype.** Imago: CNU–TER–BU–2018206 (Fig. 3), deposited in CNU, a well-preserved alate but with incomplete wings.

**Paratypes.** All termites listed in Supplementary File 1. Representatives: Soldier, CNU–TER–BU–2018201(Fig. 2e–h and Supplementary Fig. 3 1h–n); Nymph, CNU–TER–BU–2018202 (Fig. 2d and Supplementary Fig. 3a, b, d and e); Worker/pseudergate, CNU–TER–BU–2018203 (Supplementary Fig. 3c, f and g).

## The oldest known stolotermitids in the eusocial colonies

The behaviours of the cooperative brood care and overlapping generations are generally carried out by colonies, which have been scarcely documented in fossil records. Of the two, cooperative brood care fundamentally ensures the accessibility of nutrition. As indigestible cellulose is the basic diet for all termites, symbiotic digestive bacteria and protozoa are essential and must be acquired. The immatures need trophallaxis and other nursing from mainly workers in the colony, to reacquire the gut bacteria and protozoa after each moulting [25–27]. The immatures preserved in CNU008418 are uniformly distributed (Fig. 1a) among the workers/pseudergates and soldiers, suggesting a close mutual nursing relationship conforming to advanced cooperative brood care, in contrast to the primitive biparental care (without brood care from the same generation) found from *Cryptocercus* [28]. Furthermore, regarding the reproductive parents of the neonates of different instars, the preserved soldiers or workers/pseudergates are unlikely, since they do not show a developed reproductive system as documented for the fertile soldiers in *Zootermopsis nevadensis* [29]. This suggests the reproductive parents are queen and king, but they are not preserved in the amber piece. The preserved sterile adults, including soldiers and workers/pseudergates and the immatures of continuous instars that can be recognized as a generation, in conjunction with their parents (queen and king not preserved in the amber piece), form the overlapping generations in the colony. In summary, the colony fragment of CNU008418 not only preserves the morphological specialization, but also logically preserves the cooperative brood care and overlapping generations of the eusocial lifestyle of the *Cosmotermes multus***gen. et sp. nov.**

**Figure 4.**
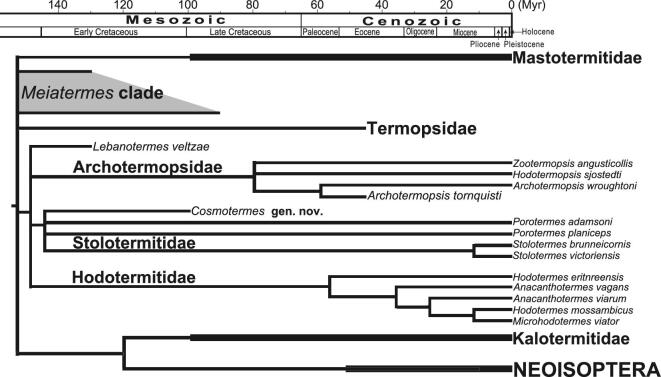
Phylogenetic position of *Cosmotermes***gen. nov.** in Stolotermitidae. The phylogenetic tree is based on the strict consensus.

The two new species of *Cosmotermes***gen. nov.** are preliminarily assigned to Hodotermitidae sensu lato (Archotermopsidae + Stolotermitidae + Hodotermitidae) [17] based on the following characters: ocelli absent, sterile castes with vestigial compound eyes, soldier mandibles decurved from base to apex. Furthermore, head flat, subsidiary tooth present, tibial spur formula 3–3–2, tarsi tetramerous and the straight basal suture are applied to assign *Cosmotermes* into Stolotermitidae according to the diagnosis [30]. The three castes of *Cosmotermes* have similar external morphologies compared to extant Stolotermitidae. However, some features in *Cosmotermes* are intermediate between *Stolotermes* (Stolotermitinae) and *Porotermes* (Porotermitinae), such as the 3–3–2 tibial spur formula consistent with *Stolotermes* but femur not swollen consistent with *Porotermes*. Besides, the mandible marginal teeth of *Cosmotermes* are obviously sharper compared with the obtuse marginal teeth of known stolotermitids. Fossil termites of Stolotermitidae are scarce, even non-existent hitherto in the Cretaceous [30–32]. The oldest stolotermitid fossil in the literature is a forewing of *Chilgatermes diamatensis*, with an age of 28–27 Ma (Early Chattian) [33]. Such a time is much later than the estimated divergence time of Stolotermitidae about 98–133 Ma, which was calculated from molecular phylogenetic analyses and a molecular clock [4]. Results of phylogenetic analysis in this study (see Supplementary File 2 for the matrix and see Supplementary Text for particulars of the phylogenetic analysis) confirm the phylogenetic position of *Cosmotermes* is at the base of Stolotermitidae (Fig. 4 and Supplementary Figs 4–6), forming a paraphyly together with *Porotermes*. These 100-million-years oldest known stolotermitids are consistent with the estimated age range of the divergent time of Stolotermitidae [4]. With the new genus added, four genera with 15 species are classified in Stolotermitidae (Table 1). The known extant stolotermitids mainly live in the southern hemisphere with records from Africa, South America and Australasia; they dwell in dead dampwood with sufficient moisture and construct tunnels in the stumps, logs, even the root system [33].

**Table 1.** Reported species of Stolotermitidae.

Species	Type	Distribution
†*Cosmotermes multus* Zhao, Yin, Shih and Ren **gen. et sp. nov.**	Fossil (mid-Cretaceous)	Myanmar
†*Cosmotermes opacus* Zhao, Yin, Shih and Ren **sp. nov.**	Fossil (mid-Cretaceous)	Myanmar
†*Chilgatermes diamatensis* Engel, Pan and Jacobs, 2013	Fossil (Oligocene)	Ethiopia
*Porotermes quadricollis* Rambur, 1842	Extant	Argentina, Chile
*Porotermes adamsoni* Froggatt, 1897	Extant	Australia, New Zealand
*Porotermes planiceps* Sjöstedt, 1904	Extant	South Africa
†*Stolotermes amanoi* Fujiyama, 1983	Fossil (Miocene)	Japan
†*Stolotermes kupe* Kaulfuss, Harris and Lee, 2010	Fossil (Miocene)	New Zealand
*Stolotermes brunneicornis* Hagen, 1858	Extant	Australia
*Stolotermes ruficeps* Brauer, 1865	Extant	Australia
*Stolotermes australicus* Mjöberg, 1920	Extant	Australia
*Stolotermes queenslandicus* Mjöberg, 1920	Extant	Australia
*Stolotermes victoriensis* Hill, 1921	Extant	Australia
*Stolotermes africanus* Emerson, 1942	Extant	South Africa
*Stolotermes inopinus* Gay, 1969	Extant	New Zealand

†symbol means the extinct species

## Palaeoecological study of *Cosmotermes* base on taphonomic evidence

In the CNU008418, 89 preserved individuals, even though they are only a portion of the colony, provided meaningful data for the ratio of the different castes and instars. A large percentage (28%) are the immatures of different instars, suggesting a rapidly growing population in various instars. Besides, no alates or nymphs are present in CNU008418. Such a constitution is consistent with the termite young colonies in the starting years [26]. The ratio of different termite castes in the colonies, regulated by extrinsic factors (mainly pheromones) and genetic inheritance [34,35], are diverse among different species. The average percentage of soldiers of *Cosmotermes multus***gen. et sp. nov.** is 10.5% (CNU008418 + CNU008281), on the high end of the recorded observation range in Hodotermitidae sensu lato from 1% to 10.2% [36,37]. The higher soldier percentage in the young colonies were either inherent or to provide defensive needs under environmental stress.

**Figure 5.**
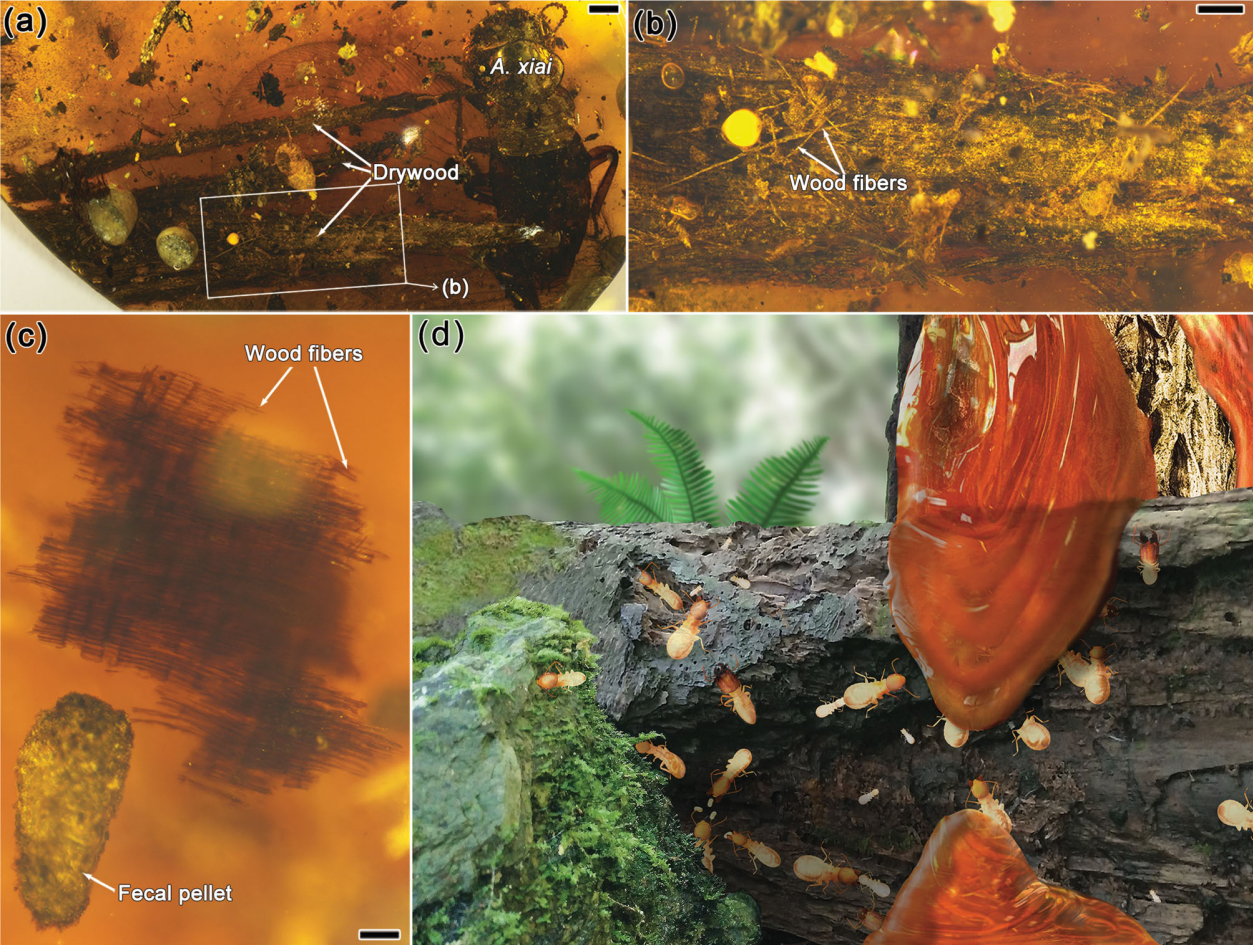
Woods in Burmese amber. (a) A piece of drywood preserved with a soldier of *Anisotermes xiai* in CNU008296. (b) Detail of the drywood. The dry fibres are relatively furcate and opaque. (c) Detail of the dampwood fragment preserved with *Cosmotermes multus***gen. et sp. nov.** in CNU008418. The fibres are plump, hyaline and well bonded. (d) Ecological reconstruction of the mid-Cretaceous dampwood termite, *Cosmotermes multus***gen. et sp. nov.** Scale bars: 1 mm in (a); 0.5 mm in (b); 0.1 mm in (c).

Termitophilous rove beetles with specialized morphologies have also been documented from mid-Cretaceous Burmese amber [38,39], raising the question of which termite or ant species might have been their host, while the known termite hosts reported for Trichopseniini are only in the basal termites Mastotermitidae and Kalotermitidae. Although the termite eusocial aggregations reported in this study verify the existence of termite societies, we cannot suggest *Cosmotermes***gen. nov.** within the family Stolotermitidae as potential hosts in the Cretaceous, since reported observations indicate rove beetles have never been reported in the nests of known stolotermitids.

Termites basically dwell in three types of nests: subterranean, drywood and dampwood. For the wood-dwelling termites, the wood source and the exterior of the fecal pellets are corresponding. The fecal pellets of drywood termites generally have six lateral surfaces separated by six longitudinal ridges due to the action of the rectum [40,41]. However, dampwood termites’ fecal pellets have a relatively irregular shape without ridges because of the water-rich wood source [42]. The irregular fecal pellets preserved with *Cosmotermes multus* are recognizable as those from other dampwood termites. The fecal pellets of *Cosmotermes opacus***sp. nov.** are mostly rice-shaped but without obvious ridges.

In addition, a small piece of dampwood is present in CNU008418 (Figs 1i and 5c), close to the *Cosmotermes multus* individuals in the same layer and a mass of wood/frass debris can be found with the same species in CNU008281, indicating co-embedded taphonomic evidence. On the contrary, drywood preserved in Burmese amber has significant morphological differences from the dampwood fragment. A piece of drywood preserved with a soldier of *Anisotermes xiai* (CNU–TER–BU–2017003) [17] has rough and opaque wood fibre (Fig. 5a and b) in contrast to the relatively hyaline and moist wood fibre preserved in CNU008418 (Fig. 5c). With the aforementioned evidence and arguments, we conclude that eusocial *Cosmotermes multus* most probably nested in damp/rotting wood and this kind of habitat environment was also adopted by their extant stolotermitid relatives. A graphical reconstruction (Fig. 5d) is shown to portray the ecology of the mid-Cretaceous dampwood termite, *Cosmotermes multus*.

## METHODS

### Specimen preparation

All the specimens involved are housed at the CNU. The four amber specimens were identified from our amber termite collection, which mostly comprises imagoes. Although alate aggregations also exist, they are unpersuasive to explain anything about eusocial gregariousness, so we aim at the studies of non-reproductive termites. After incising and polishing, the amber specimens were observed, measured and photographed using a Nikon SMZ25 microscope system. The photos were stacked using Helicon Focus. Simplified drawings were prepared using Adobe Illustrator CC and further rendered using Adobe Photoshop CC.

### Phylogenetic analysis

The phylogenetic analysis was conducted to clarify the phylogenetic position of the new genus in the evolution of basal termites. The matrix (Supplementary Files 2 and 3) was set up using the one from our previous study [17] with the addition of the new taxon. The taxa were filtered with at least body structure preserved. The two new species have only one different character and therefore operated together as *Cosmotermes* with a compound value of character #21 (the length of the metabasitarsomere). The analysis in TNT v1.5 [43] was conducted by using ‘New Technology Search’ with Sectorial search, Ratchet, Drift and Tree fusing analyses. The minimum length was set to be found 100 times. The consensus trees are using strict consensus (Fig. 4 and Supplementary Figs 4 and 6), Bremer support (Supplementary Fig. 4) and majority rules consensus (Supplementary Fig. 5). A repeated analysis was run in WINCLADA v1.00.08 [44] using NONA and set to keep 10 000 maximum trees, 1000 replications and 100 starting trees per replication. The consensus tree is using strict consensus with characters mapped (Supplementary Fig. 6).

## Supplementary Material

nwz141_Supplemental_Files
