# Supplementary material for: Termite colonies from mid-Cretaceous Myanmar demonstrate their early eusocial lifestyle in damp wood
Source: Natl Sci Rev. 2019 Sep 13;7(2):381–90. doi: 10.1093/nsr/nwz141 (PMC8288961; doi:10.1093/nsr/nwz141)
Supplement: nwz141_Supplemental_Files [file nwz141_supplemental_files.zip › Supplementary_figures_and_text.docx]

**Supplementary Online Content**

**Termite colonies from mid-Cretaceous Myanmar demonstrate their early eusocial lifestyle in damp wood**

Zhipeng Zhao, Xiangchu Yin, Chungkun Shih, Taiping Gao* & Dong Ren*

**Supplementary Result.** Systematic palaeontology and Phylogenetic results.

**Supplementary Figure S1.** *Cosmotermes multus* gen. et sp. nov. in CNU008418.

**Supplementary Figure S2.** Details captured from the eusocial gregariousness in CNU008418.

**Supplementary Figure S3.** *Cosmotermes opacus* sp. nov., nymph (CNU–TER–BU–2018202), worker/pseudergate (CNU–TER–BU–2018203) and soldier (CNU–TER–BU–2018201).

**Supplementary Figure S4.** Strict consensus tree calculated from TNT.

**Supplementary Figure S5.** Majority rule (50%) consensus tree calculated from TNT.

**Supplementary Figure S6.** Strict consensus tree calculated from Winclada.

**Supplementary File 1.** Data of the termites in the three aggregations. (.xls)

**Supplementary File 2.** Matrix and characters used in the phylogenetic analyses. (.xls)

**Supplementary File 3.** Matrix using in the phylogenetic analyses. (.tnt)

**Systematic palaeontology**

Order Blattodea, Brunner von Wattenwyl, 1882

Epifamily Termitoidae, Latreille, 1802

Family Stolotermitidae, Engel, Grimaldi and Krishna, 2009

Genus *Cosmotermes* Zhao, Yin, Shih & Ren **gen. n.**

**Diagnosis.** *Imago,* head rounded dorsally, flat laterally; clypeus hyaline; mandibles not exceeding clypeus; left mandible with one apical tooth and three marginal teeth; right mandible with one apical tooth and two marginal teeth, subsidiary tooth present between apical tooth and first marginal tooth; ocelli absent; compound eyes well-developed, with relatively big size, rounded in lateral view, locating in the middle of head lateral margin, without anterior emargination; antenna moniliform, with about 19 articles; Y-suture present as a groove; pronotum with width slightly shorter than head, anterior margin convex, lateral margin evenly converging posteriorly, posterolateral corners arched, posterior margin relatively straight but with a centraxonial groove; wings slightly reticulated, with C, R and Rs more sclerotized and pigmented than M and Cu; C not uniting with costal margin, but terminating together with the end of Rs, not fading away compare with majority termites. Sc and R short and simple; Rs width moderate, with about seven branches, terminating anteriorly to wing apex. Medial field covering wing apex, with four branches; CuA long, terminating on posterior margin, with 6 branches; CuP vestigial, terminating on the basal suture apart from posterior margin; basal suture straight on forewings; tibial spines absent; tibial spur formula 3–3–2; tarsi tetramerous; arolium absent; cerci thin and short, with about five articles. *Soldier*, head oval in dorsal view, flat in lateral view, much longer than wide; clypeus long and hyaline; mandibles relatively short, taking up about 1/3 length of head, with incisive teeth, decurved from stem to apex; left mandible with one apical tooth and three marginal teeth, right mandible with two marginal teeth; antenna moniliform and plump, with 16–20 articles; compound eyes present, locating posterior to antennal socket, small and rudimentary; ocelli absent; pronotum flat, with width distinctly narrower than head, anterior margin convex, anterolateral corners projecting, lateral margin evenly converging posteriorly, posterior margin straight; mesonotum and metanotum lateral margin converging anteriorly, posterior margin relatively straight; tibial spines absent; tibial spur formula 3–3–2; tarsi tetramerous; arolium absent. *Worker/Pseudergate*, head rounded in dorsal view, flat in lateral view; clypeus short; mandible dentition according with imago; compound eyes present with anterior emargination; ocelli absent; tibial spines absent; tibial spur formula 3–3–2; tarsi tetramerous; arolium absent.

*Cosmotermes multus* Zhao, Yin, Shih & Ren **gen. et sp. nov.**

**Description**

***Holotype*.** CNU–TER–BU–2018077 (Fig. 1B). *Soldier,* body unhairy; exoskeleton mostly hyaline except the strongly sclerotized mandible teeth; body length 5.05 mm in total; head oval in dorsal view, length 2.51 mm, width 1.35 mm; labrum shorter than mandibles, trapezoidal, length 0.36 mm, width 0.42 mm; mandibles gently occlusal, decurved from stem to apex; left mandible length 0.84 mm, with one apical tooth and three marginal teeth, first marginal tooth toward lateral anteriority, the other two toward lateral; right mandible length 0.78 mm, with a apical tooth and two marginal teeth, a lateral notch present near the first marginal tooth; antennae moniliform, both left and right with 12 articles preserved; compound eyes small and vestigial, with anterior shrink, diameter 0.15 mm in dorsal view; Y-suture on head invisible; pronotum width 0.91 mm, centraxonial length 0.5 mm, with width distinctly narrower than head, anterior margin convex, anterolateral corners projecting, lateral margin evenly converging posteriorly, posterior margin straight; abdomen flat with partial intestine visible; styli and cerci invisible in dorsal view.

***Paratype*.** CNU–TER–BU–2018005 (Fig. 1E). *Worker/pseudergate,* body unhairy; exoskeleton mostly hyaline. Body length 4.82 mm with mandibles occlusal; head rounded in dorsal view, length 1.55 mm from labrum anterior margin to head posterior margin, width 1.32 mm; labrum much shorter than length, width 0.46 mm; mandibles gently occlusal, decurved from stem to apex; left mandible length 0.37 mm along teeth, with one apical tooth and three sharp marginal teeth, molar plate with numbers of fine ridges; right mandible length 0.38 mm, with one apical tooth and two marginal teeth, a subsidiary tooth present between apical tooth and first marginal tooth; antennae moniliform, with 17 articles; compound eyes moderate size, with anterior shrink, diameter 0.19 mm in dorsal view; Y-suture absent; pronotum width 1.09 mm, centraxonial length 0.6 mm, with width narrower than head, anterior margin convex, anterolateral corners projecting, lateral margin evenly converging posteriorly, posterior margin straight; tibial spur formula 3–3–2; abdomen flat with partial intestine visible; styli and cerci invisible in dorsal view.

***Paratype*.** CNU–TER–BU–2018101 (Fig. 2B). *Soldier,* body unhairy; exoskeleton mostly hyaline except the strongly sclerotized mandible teeth; body length 4.41 mm; head oval in dorsal view, length 2.19 mm, width 1.26 mm; mandibles occlusal, decurved from stem to apex; left mandible with one apical tooth and three marginal teeth; right mandible with a apical tooth and two marginal teeth, a lateral notch present near the first marginal tooth; mandibular muscle well developed; antennae moniliform, with 16 articles; vestigial compound eyes present posterior to antennal sockets; pronotum 0.86 mm, centraxonial length 0.43 mm; mesonotum 0.81 mm, metanotum 0.94 mm; tibial spur formula 3–3–2;

*Cosmotermes opacus* Zhao, Yin, Shih & Ren **sp. nov.**

**Description**

***Holotype*.** CNU–TER–BU–2018206 (Fig. 3). *Imago,* whole body chestnut colour, opaque expect pronotum and labrum; antenna moniliform, with about 19 articles; body length 5.9 mm excluding the wings; head length 1.83 mm, width 1.43 mm; labrum length 0.43 mm, width 0.56 mm, with sparse setae in anterior margin; compound eyes plump, diameter 0.38 mm; pronotum centraxonial length 0.69 mm, width 1.34 mm; wing length 7.8 mm, width 2.41 mm; Sc short, terminating in basal quarter of wing length; R terminating at about basal third of wing length near wing base; Rs width moderate, taking up a quarter width of the wing, with 7 branches, terminating anteriorly to wing apex. medial field covering wing apex, with four branches; CuA terminating in apical third of wing length; CuP vestigial, terminating on the basal suture apart from lower margin; basal suture straight on forewings. basitarsomere length equal to width in fore-leg, about twice to width in mid-leg, about 3 times to width in hind-leg; tibial spur formula 3–3–2; tarsi tetramerous; arolium absent; cerci thin and short, with 5–6 cercomeres.

***Paratype*.** CNU–TER–BU–2018202 (Figs. 2D and S3A, B, D, E). *Nymph,* exoskeleton opaque yellow; body incompletely preserved, length more than 4.59 mm; head rounded, width 1.32 mm, length 1.55 mm; mandibles occlusive, not exceeding to labrum, teeth invisible; Y-suture present; antenna wizened, with 19 articles; compound eyes present, vestigial and small, with anterior emargination; pronotum shape similar to that of imago, width 1.05 mm; mesonotum width 1.05 mm, metanotum width 1.03 mm; wing buds present on the posterolateral corners of mesonotum and metanotum; right fore-leg and mid-leg visible both with 3 tibial spurs, spines along tibiae absent; abdomen plump with anterior four segments partial preserved.

***Paratype*.** CNU–TER–BU–2018201 (Figs. 2E–H and S3H–N). *Soldier,* exoskeleton chestnut colour, opaque expect nota and labrum. Body length 6.95 mm with mandibles opened; head oval in dorsal view, length 2.75 mm, width 1.3 mm; labrum shorter than mandibles, trapezoidal, length 0.36 mm, width 0.42 mm; mandibles decurved from stem to apex; left mandible length 0.84 mm, with one apical tooth and three marginal teeth; right mandible length 0.78 mm, with an apical tooth and two marginal teeth; antennae moniliform, both left and right with 12 articles preserved; compound eyes small and vestigial, with anterior emargination, diameter 0.15 mm in dorsal view; Y-suture distinct; pronotum width 1.02 mm, centraxonial length 0.65 mm, with width distinctly narrower than head, anterior margin convex, anterolateral corners projecting, lateral margin evenly converging posteriorly, posterior margin straight; tibial spur formula 3–3–2; abdomen spindly; styli present; cerci present but cercomeres uncountable.

**Phylogenetic results**

The search in TNT returned: 36 most parsimonious trees retained with the score= 268. All the 36 most parsimonious trees were using to construct the strict consensus tree (Fig. S4), majority rule (Fig. S5).

The search in Winclada returned: 25 most parsimonious trees with 268 steps. All the 25 equally most parsimonious trees were using to construct the strict consensus tree with characters mapped (Fig. S6). The topology of the strict consensus is same with the result calculated from TNT.

The strict consensus (Figs. S4, 6) is basically same with the results from our previous study beside the newly added taxon located in Stolotermitidae. The monophyly of each families is fine, while the resolution for the families is not quite sharp, may because of the evolutionary radiation. The majority rule consensus tree (Fig. S5) with higher resolution may give a better topology in accord with the order of cladistics.

**
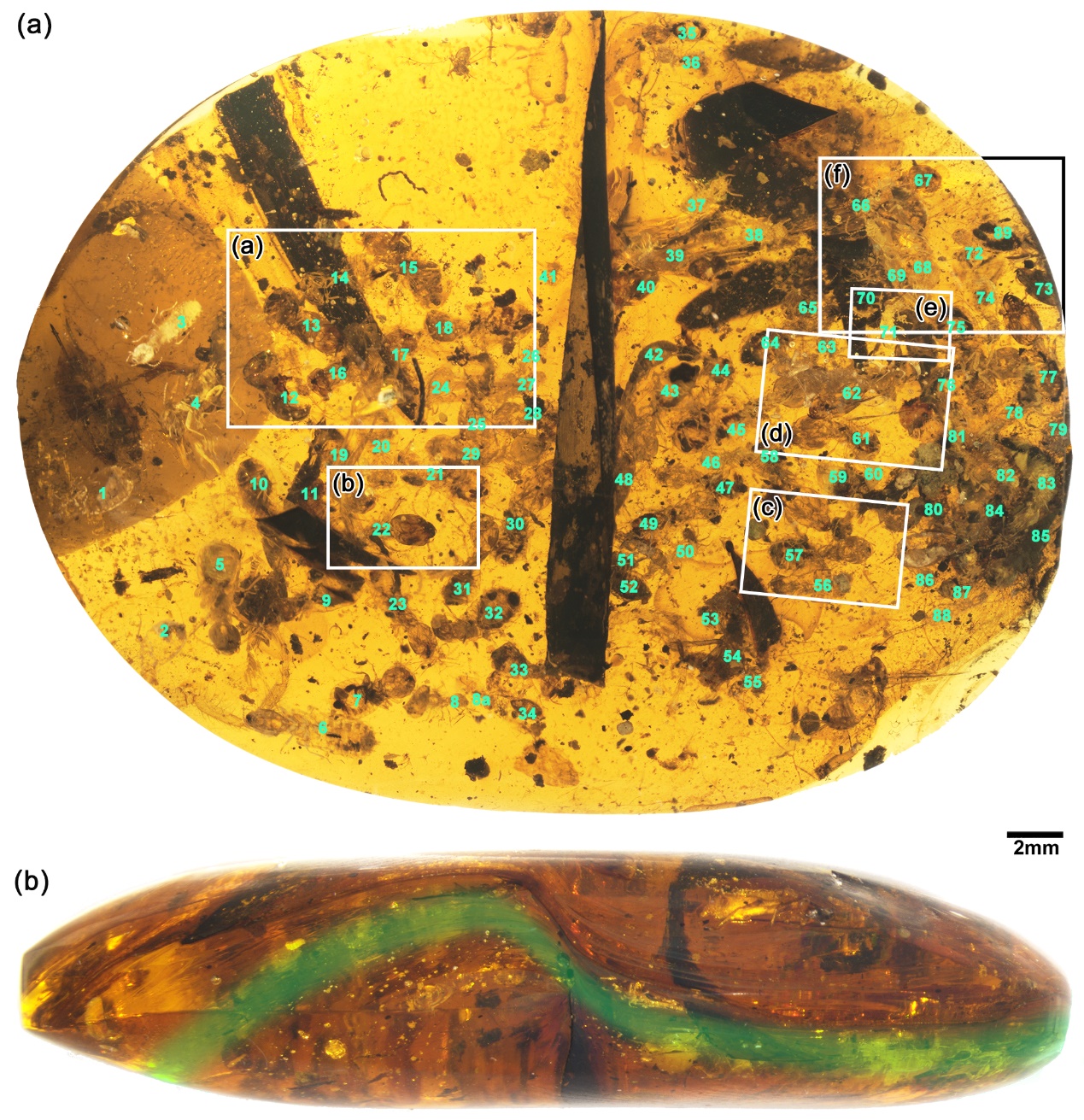
 Supplementary Figure S1.** *Cosmotermes multus* gen. et sp. nov. in CNU008418. (a) The termite specimen numbers are constituted with CNU–TER–BU–20180+”nn” (nn represents the double-digit number in the Figure). The details in boxes are enlarged and shown in Fig. S2. (b) Profile view. All 89 termites, fecal pellets and the wood fragment are preserved on the internal curved layer marked with green colour.


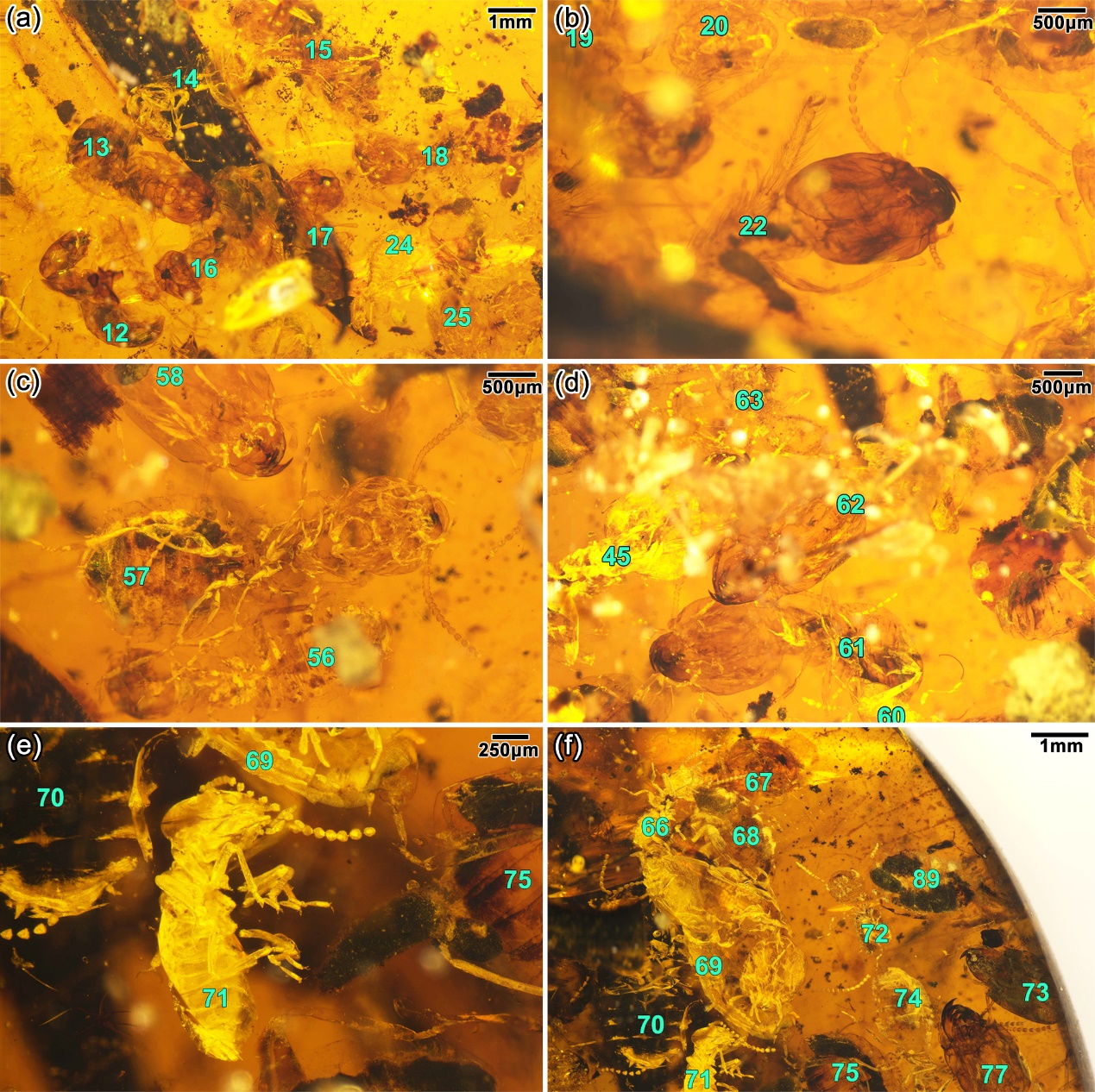


**Supplementary Figure S2.** Details captured from the eusocial gregariousness in CNU008418. The scopes are marked in Fig. S1.

**
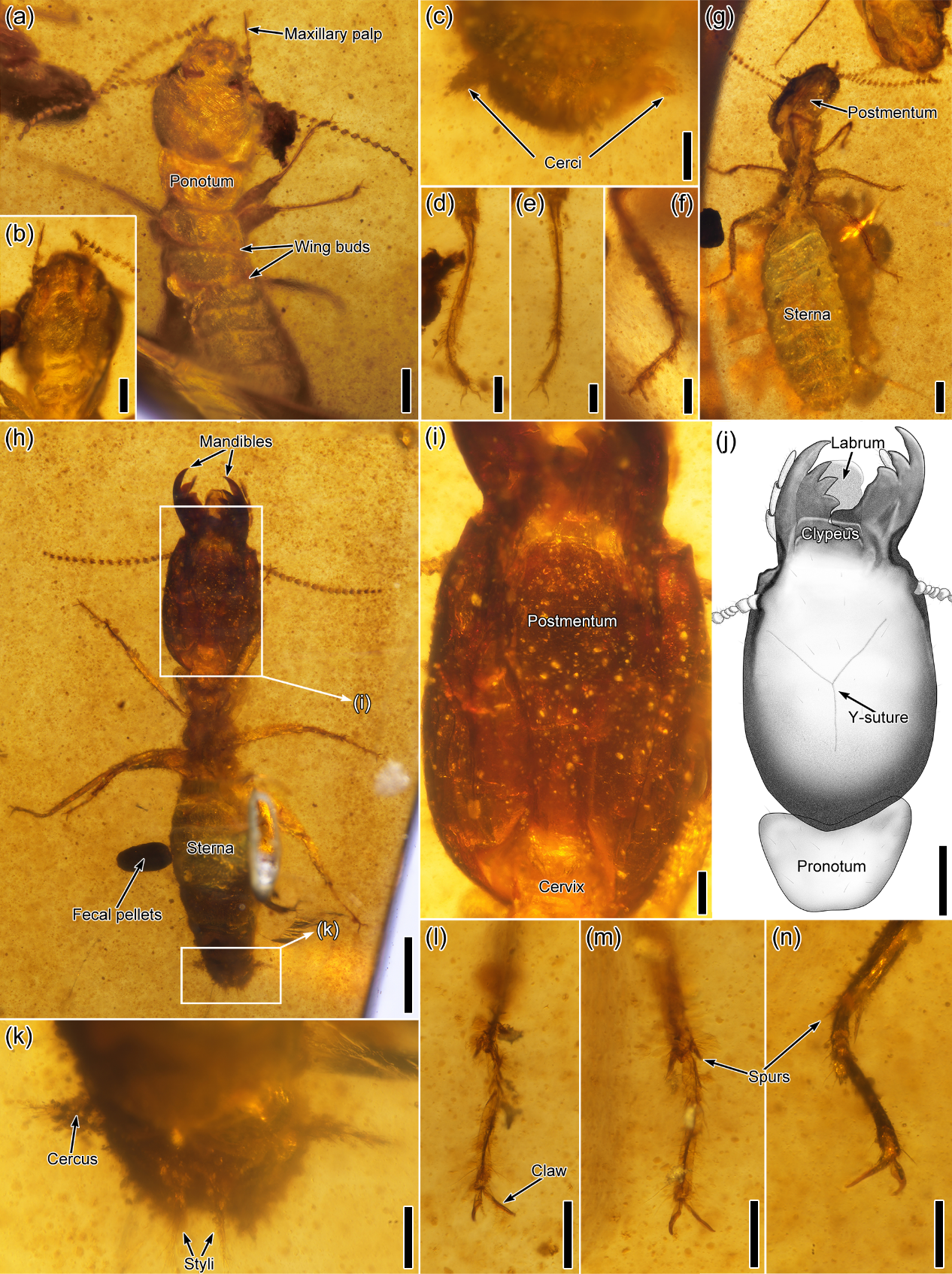
**

**Supplementary Figure S3.** *Cosmotermes opacus* sp. nov., nymph (CNU–TER–BU–2018202), worker/pseudergate (CNU–TER–BU–2018203) and soldier (CNU–TER–BU–2018201). (a), (b), (d) and (e) Nymph. (a) Dorsal habitus. (b) Head in ventral view. (d) and (e) Fore-leg and mid-leg. (c), (f) and (g) Worker/pseudergate. (c) Postabdomen in ventral view. (f) Hind-leg. (g) Ventral habitus. (h) – (n) Soldier. (h) Ventral habitus. (i) Head capsule in ventral view. (j) Drawing of head and pronotum. (k) Postabdomen. (l) – (n) Fore-leg mid-leg and hind-leg with tibiae, tarsi and claws. Scale bars: 1 mm in (h); 0.5 mm in (a), (g) and (j); 0.2 mm in others.


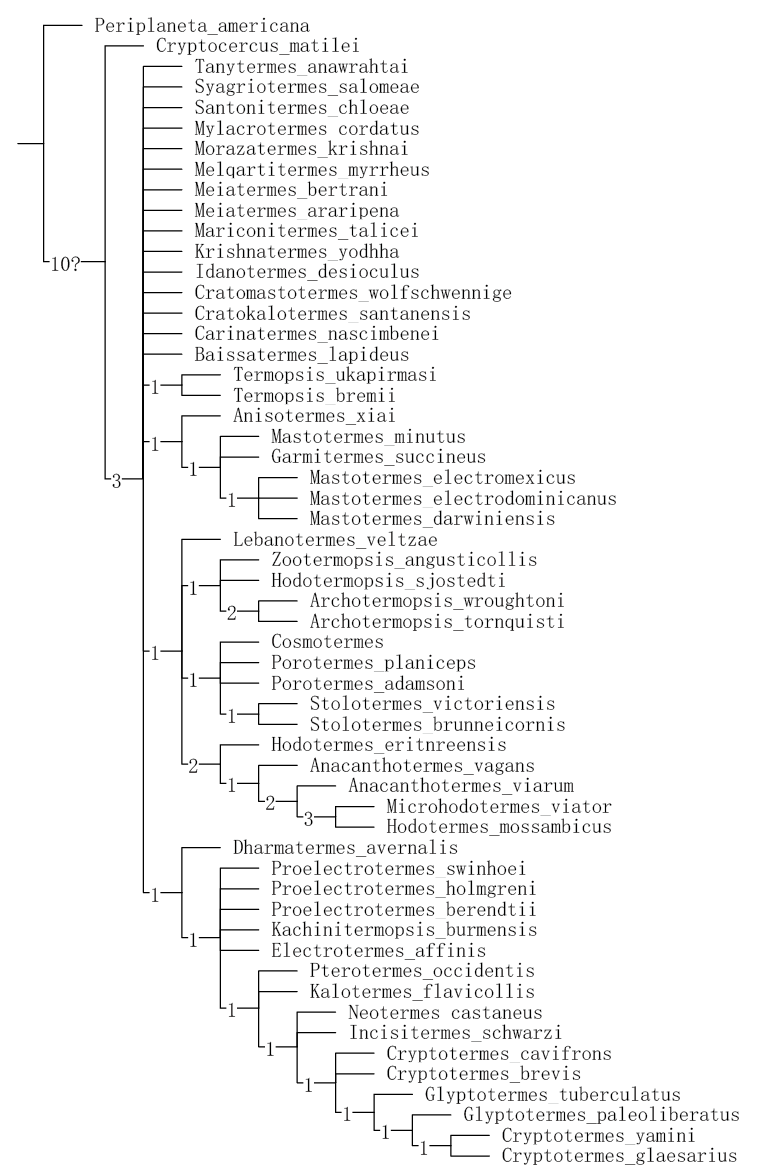


**Supplementary Figure S4.** Strict consensus tree calculated from TNT. Numbers on the nodes represent the Bremer support value.


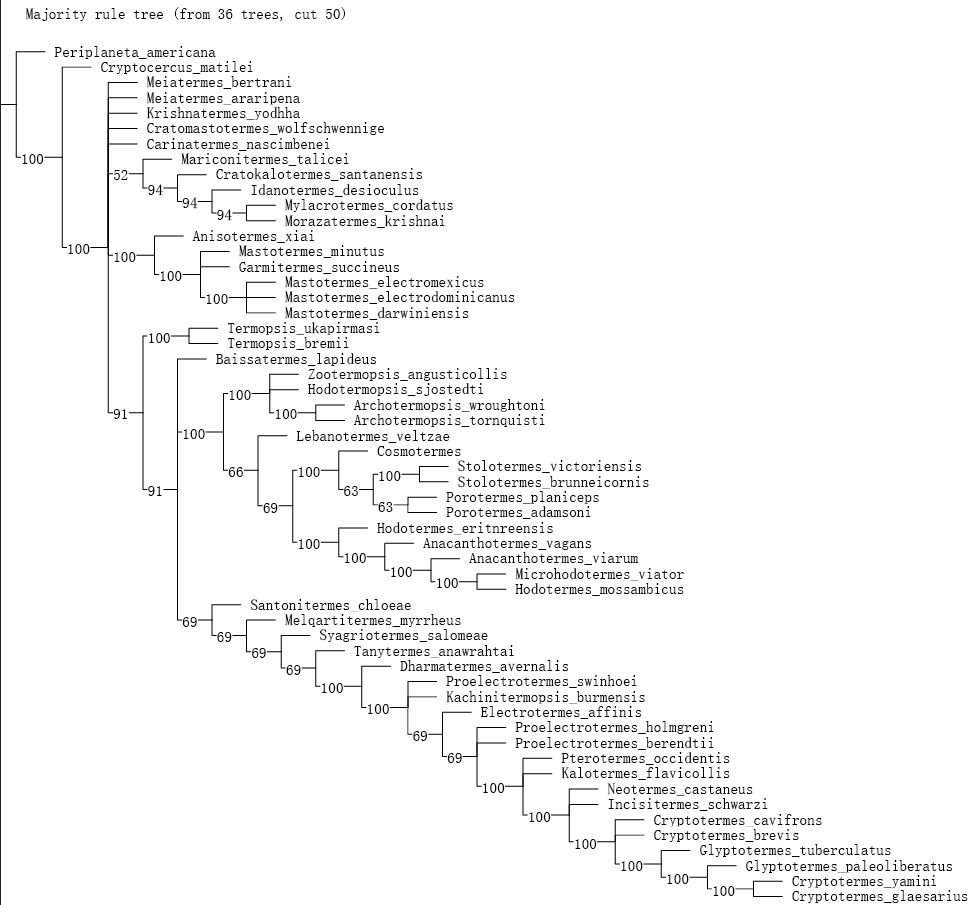


**Supplementary Figure S5.** Majority rule (50%) consensus tree calculated from TNT. Nodal values represent the percentage of occurrence of the nodes in all 36 equally most parsimonious trees.


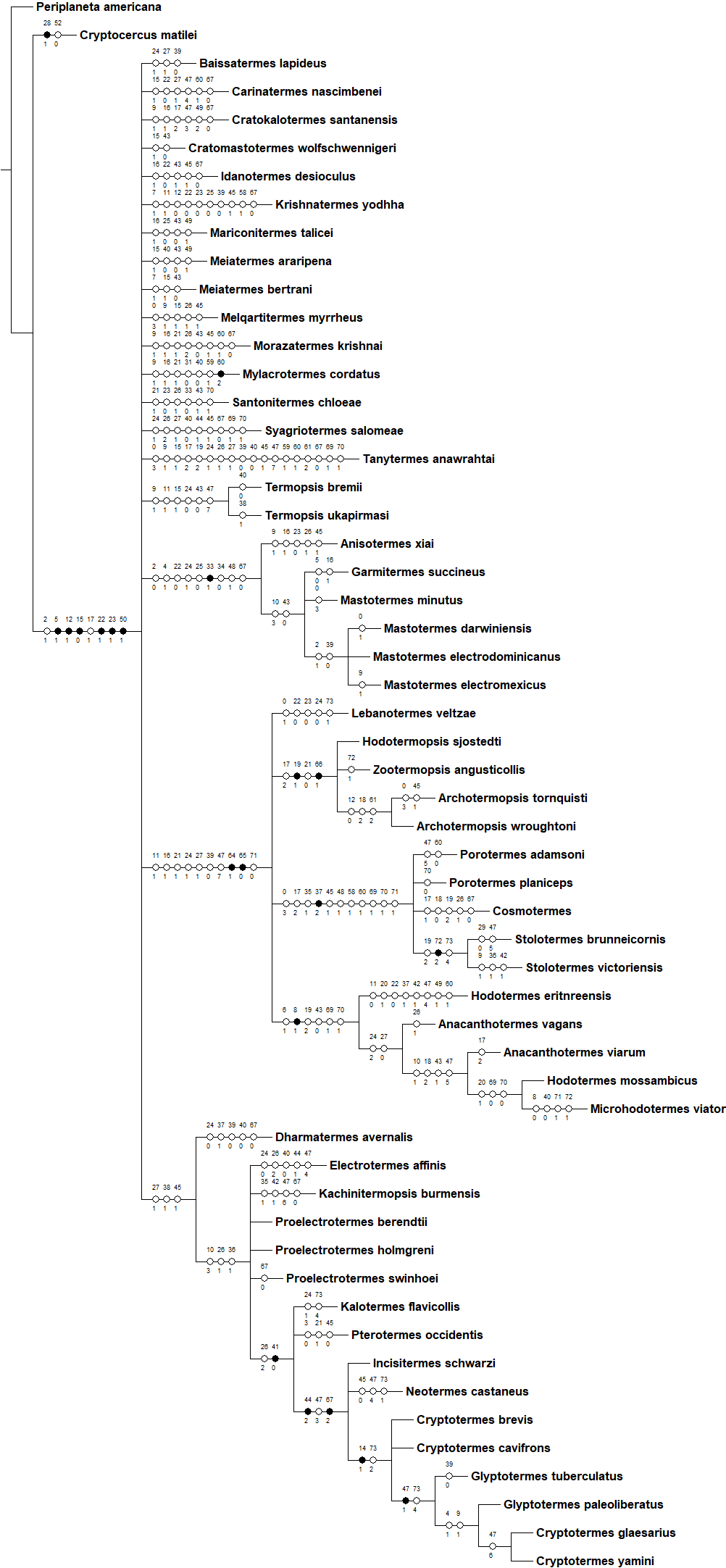


**Supplementary Figure S6.** Strict consensus tree calculated from Winclada. The black hashmark represent the synapomorphies and the white hashmark represent the plesiomorphies. Please note the characters numbering starts from zero in the tree but the matrix from one)
